# Highly porous non-precious bimetallic electrocatalysts for efficient hydrogen evolution

**DOI:** 10.1038/ncomms7567

**Published:** 2015-03-16

**Authors:** Qi Lu, Gregory S. Hutchings, Weiting Yu, Yang Zhou, Robert V. Forest, Runzhe Tao, Jonathan Rosen, Bryan T. Yonemoto, Zeyuan Cao, Haimei Zheng, John Q. Xiao, Feng Jiao, Jingguang G. Chen

**Affiliations:** 1 Center for Catalytic Science and Technology, Department of Chemical and Biomolecular Engineering, University of Delaware, Newark, Delaware 19716, USA; 2 Department of Chemical Engineering, Columbia University, New York, New York 10027, USA; 3 Department of Physics and Astronomy, University of Delaware, Newark, Delaware 19716, USA; 4 Materials Sciences Division, Lawrence Berkeley National Laboratory, Berkeley, California 94720, USA; 5 Department of Mechanical Engineering, University of Delaware, Newark, Delaware 19716, USA

## Abstract

A robust and efficient non-precious metal catalyst for hydrogen evolution reaction is one of the key components for carbon dioxide-free hydrogen production. Here we report that a hierarchical nanoporous copper-titanium bimetallic electrocatalyst is able to produce hydrogen from water under a mild overpotential at more than twice the rate of state-of-the-art carbon-supported platinum catalyst. Although both copper and titanium are known to be poor hydrogen evolution catalysts, the combination of these two elements creates unique copper-copper-titanium hollow sites, which have a hydrogen-binding energy very similar to that of platinum, resulting in an exceptional hydrogen evolution activity. In addition, the hierarchical porosity of the nanoporous copper-titanium catalyst also contributes to its high hydrogen evolution activity, because it provides a large-surface area for electrocatalytic hydrogen evolution, and improves the mass transport properties. Moreover, the catalyst is self-supported, eliminating the overpotential associated with the catalyst/support interface.

## Abstract (web summary)

Investigations into non-precious metal catalysts for hydrogen evolution are ongoing. Here, the authors report a hierarchical, nanoporous copper-titanium electrocatalyst, and demonstrate that it catalyses hydrogen production at twice the over-all rate of commercial platinum-based catalysts.

## Graphical abstract

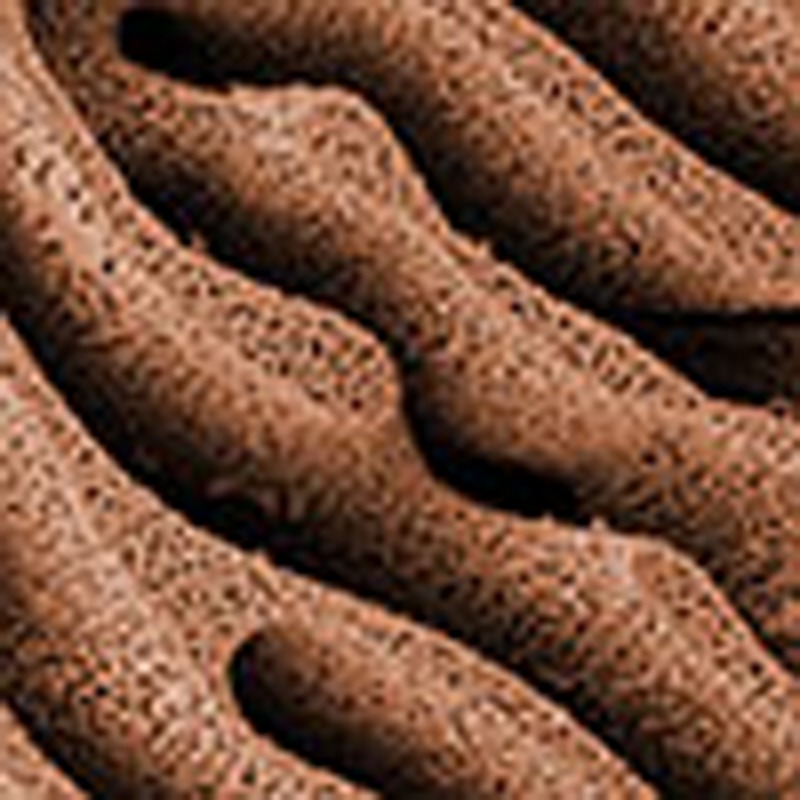

The rising concerns about CO_2_ emissions have led to a growing realization that it is not possible to sustain the world’s current development based on fossil fuels without a substitution of clean and renewable energy^1^,^2^,^3^. Hydrogen, other than being an important chemical feedstock in global industry, is now firmly considered as one of the most likely future fuels^4^,^5^. However, current hydrogen production primarily relies on the steam methane reforming process which is neither sustainable nor favoured because the process requires high-energy (heat) input and produces CO_2_ as a by-product^5^,^6^,^7^. It is widely believed that room temperature electrochemical reduction of water to molecular hydrogen offers a significant promise for supplying CO_2_-free hydrogen, which can be used directly as a fuel or as reactant to convert CO_2_ and to upgrade petroleum and biomass feedstocks to value-added chemicals and fuels through hydrotreating processes^8^,^9^,^10^. All these applications require large-scale, commercial processes for water electrolysis, which in turn require breakthrough discoveries in at least two areas: (i) the availability of electricity derived from renewable energy sources, such as solar and wind, and (ii) the discovery of low-cost electrocatalysts to replace precious metals that are currently the state-of-the-art hydrogen evolution reaction (HER) catalysts.

HER in an acidic environment generally requires lower overpotentials than those in a basic environment^11^,^12^,^13^. However, a hydrogen production system in a basic environment is still more promising, because of the possibility to consider non-precious-metal-based catalysts that cannot be used in acidic conditions, not only for HER at cathode, but also for oxygen evolution reaction at anode^14^,^15^. Regardless of acidic or basic conditions, Pt, along with its alloys, is the benchmark electrocatalyst that requires very small overpotentials to drive the reaction, whereas the scarcity and high cost of Pt hinder its large-scale use for H_2_ production. As a result, enormous research efforts have been devoted to finding and engineering low-cost alternative catalysts. For example, tungsten and molybdenum carbides and sulfides^16^,^17^,^18^,^19^,^20^,^21^,^22^,^23^,^24^,^25^, nickel phosphides^26^ and electrodeposited Ni-Cu alloy^27^ have been identified as potential electrocatalysts for HER, unfortunately most of these catalysts exhibit poor intrinsic activity and/or stability in strong bases.

Over the past decade, density functional theory (DFT) predictions, in conjunction with experimental efforts, have played a pivotal role in providing design principles of electrocatalysts^28^,^29^,^30^,^31^,^32^,^33^. For hydrogen evolution, the activities (in terms of exchange current density) of different catalytic surfaces can be correlated with their hydrogen-binding energy (HBE) via a volcano-type relationship, revealing that an optimal HBE would lead to the highest activity^34^,^35^. Monometallic catalysts have been studied extensively using the DFT method. However, monometallic non-precious metals show HBE values significantly different from that of Pt. In the present paper, DFT calculations show that Cu-Ti bimetallic materials have similar HBE values as Pt, and therefore are promising non-precious metal HER electrocatalysts. These predictions are experimentally verified on both bulk Cu-Ti alloys and highly porous catalysts.

## Results

### DFT prediction of Cu-Ti bimetallic catalyst

**Figure 1:**
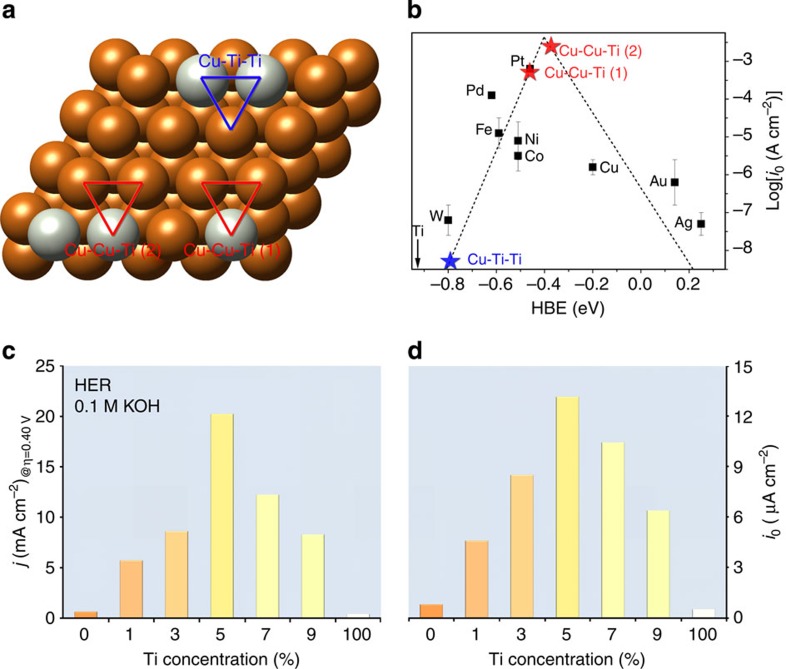
Modelling studies. (**a**) The possible bimetallic sites on a Ti-modified Cu surface. (**b**) The corresponding HBEs incorporated in a volcano plot. The error bar stands for the variation of exchange current density in different experimental measurement. A comparison of (**c**) HER activities and (**d**) exchange current densities of various bulk Cu-Ti alloy surfaces and the corresponding monometallic standards.

As monometallic catalysts, Cu and Ti are known to be poor HER catalysts because their HBE values are either too small or too large, respectively^36^. Using DFT calculations, we have demonstrated that the Cu-Cu-Ti hollow site on a Cu-Ti bimetallic surface exhibits an optimal HBE for HER. As shown in Fig. 1a, on a Ti-modified Cu surface, three distinct adsorption sites can be identified. Their corresponding HBE values were calculated using DFT and were incorporated into a volcano plot constructed from previously studied monometallic surfaces (Fig. 1b)^34^. It can be seen that the two types of Cu-Cu-Ti hollow sites exhibit HBE values very close to that of Pt. In contrast, the Cu-Ti-Ti hollow site containing two Ti atoms binds hydrogen too strongly. Therefore, replacement of one surface Cu atom with Ti on every 3 × 3 Cu unit cell would result in an optimal surface, in principle achieving a maximum density of the Cu-Cu-Ti sites without introducing the Cu-Ti-Ti-inactive sites. A lower or higher Ti content will decrease the HER activity because of insufficient number of active sites or creation of inactive sites. It should be noted that it was proposed recently that other than HBE, the binding of surface hydroxyl groups could be another descriptor of the catalytic activity for HER in base^13^,^37^. Although this finding may open a new horizon for designing HER catalyst, it also raised some different opinions regarding the effect of pH values^38^,^39^. The findings presented in this paper, however, suggest that the HBE appears to be an appropriate descriptor to predict HER activity for Cu-Ti in basic environment.

### Experimental verification of Cu-Ti bimetallic catalyst

To verify the DFT predictions, a series of Cu_100−*x*_Ti_*x*_ (*x*=1, 3, 5, 7 and 9) alloys with homogeneously distributed atoms were fabricated using an arc-melting technique followed by a melt-spinning process in order to retain their solid solution phase formed at high temperatures. After polishing, the resulting materials have smooth surfaces with roughness factor smaller than 1.1 (Supplementary Table 1). Powder X-ray diffraction (PXRD) analysis suggests all alloys retain the *fcc* structure of crystalline Cu with a lattice expansion because of Ti doping (Supplementary Fig. 1). It is well known that the elemental compositions of a bimetallic system can be different on the surface and in the bulk because the surface composition is determined as a result of minimization of alloy surface free energy with respect to atom exchange between surface and bulk^40^. X-ray photoelectron spectroscopy (XPS) characterization was therefore conducted and the results (Supplementary Fig. 2) confirmed that the surface Ti content is about twice as large as the bulk stoichiometry (Supplementary Table 1). The HER activities of all Cu-Ti alloys as well as pure Cu and Ti standards were compared by plotting their polarization curves in 0.1 M KOH electrolyte (Supplementary Fig. 3). As shown in Fig. 1c, a significant increase in HER activity can be achieved after modifying the Cu surface with as little as 1 at. % of Ti, and a maximum enhancement was observed for a bulk stoichiometry of Cu_95_Ti_5_. The surface Ti composition of Cu_95_Ti_5_ is found to be 10.9 at. %, which is in good agreement with the optimal value predicted by DFT calculations of 1 Ti atom in a 3 × 3 cell (11.1 at. %). A further increase in Ti concentration leads to a decrease of HER activity, which is likely due to the rapid formation of Cu-Ti-Ti sites resulting from the large cohesive energy of Ti.

### Design of nanostructured Cu-Ti bimetallic catalyst

To extend the DFT predictions and bulk alloy results to practical high-performance catalysts, it is important to design a nano-architecture for the catalytic material. Nanoporous materials^41^,^42^,^43^, especially recently reported nanoporous bi- and tri-metallic materials^44^,^45^ have attracted significant research interests for their enhanced electrocatalytic activities primarily due to the enhanced surface to bulk ratio. For example, a recent work reported by Kibsgaard *et al*. demonstrated that nanoporous MoS_2_ catalyst exhibited high HER activities because of its higher density of active surface sites compared with the aligned MoS_2_ nanowire counterpart^46^,^47^. However, its material utilization became worse at high reaction rates (that is, high currents), because the produced hydrogen bubbles built up inside the porous network and blocked the active sites. Here, we designed and synthesized a Cu-Ti bimetallic electrocatalyst with a highly hierarchical porous structure (denoted as np-CuTi) by making a multi-phase Al-Cu-Ti precursor (atomic ratio Al:Cu:Ti=80:19:1), followed by a dealloying process. The atomic ratio of Ti to (Cu+Ti) was chosen to be the optimal value (5 at. %) from bulk Cu-Ti studies. The nano-sized pores of the resulting np-CuTi are responsible for high surface areas, whereas the micrometre-sized pores served as gas diffusion channels to enhance mass transport properties. This catalyst is monolithic and self-supported, which enhances the electric transportation and eliminates the necessity of using a supporting conductive substrate.

**Figure 2:**
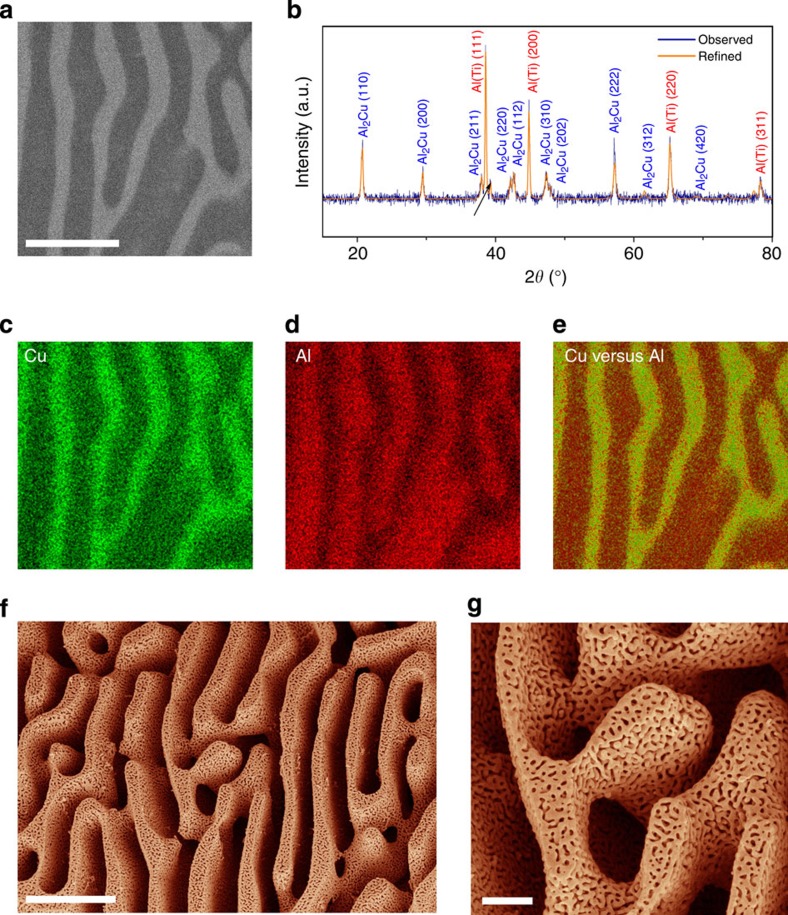
XRD and SEM characterization. (**a**) SEM image of a Al_80_Cu_19_Ti_1_ pristine catalyst electrode. Scale bar, 1 μm. (**b**) The corresponding XRD pattern. (**c**–**e**) The corresponding EDX mapping of Cu (**c**), Al (**d**) and the composite Cu versus Al (**e**). (**f**) SEM image of np-CuTi after selective dealloying. Scale bar, 1 μm. (**g**) The corresponding higher magnification SEM image. Scale bar, 200 nm.

Although the formation and catalytic application of nanoporous metals have been studied previously^48^,^49^,^50^,^51^, a highly hierarchical nanoporous bimetallic material with well-defined bimodal pore size distribution has not been explored to date. In the present study, the origin of the hierarchical porosity was explored using various structural characterizations. A typical scanning electron microscopy (SEM) image of an Al_80_Cu_19_Ti_1_ plate is presented in Fig. 2a, in which two distinctly contrasted phases were observed. The dimension of each phase, either bright or dark, is about several hundred nanometers in width and extends to several micrometers in length. Energy-dispersive X-ray spectroscopy (EDX) analysis (Fig. 2c–e) clearly shows that the bright region is a Cu-rich phase, whereas the darker region is mainly composed of Al. The location of Ti atoms cannot be determined by EDX measurements because of its low atomic concentration (1%). The PXRD pattern in Fig. 2b shows two sets of distinct diffraction profiles, corresponding to Al_2_Cu and Al. The angular positions of the indexed Al_2_Cu peaks matched the calculated values of the standardized crystal structure, whereas the Al peaks were found to be slightly shifted towards lower angles, indicating a possible crystal lattice expansion because of Ti doping. In addition, a weak Al_3_Ti peak (112; 2*θ*=39°) was also observed. The PXRD results indicate the existence of Ti in the Al-rich region in two phases: a Al-Ti solid solution phase and a metallic Al_3_Ti compound phase. The subsequent selective dealloying process conducted in strong alkaline media resulted in two different sets of pores in np-CuTi (Fig. 2f,g). The micrometre-size pores were resulted from a complete removal of the Al-rich region; the nano-sized pores were obtained by the removal of Al atoms in the Al_2_Cu compound. N_2_ adsorption-desorption measurement further confirmed that the resulting np-CuTi exhibits a relatively large Brunauer–Emmett–Teller surface area of about 46 m^2^ g^−1^ with an average nanopore size of ca 15 nm using the Barrett–Joyner–Halenda method (Supplementary Fig. 4). Note that the micrometre-sized pores in np-CuTi are too large to be measured in the gas adsorption experiments.

**Figure 3:**
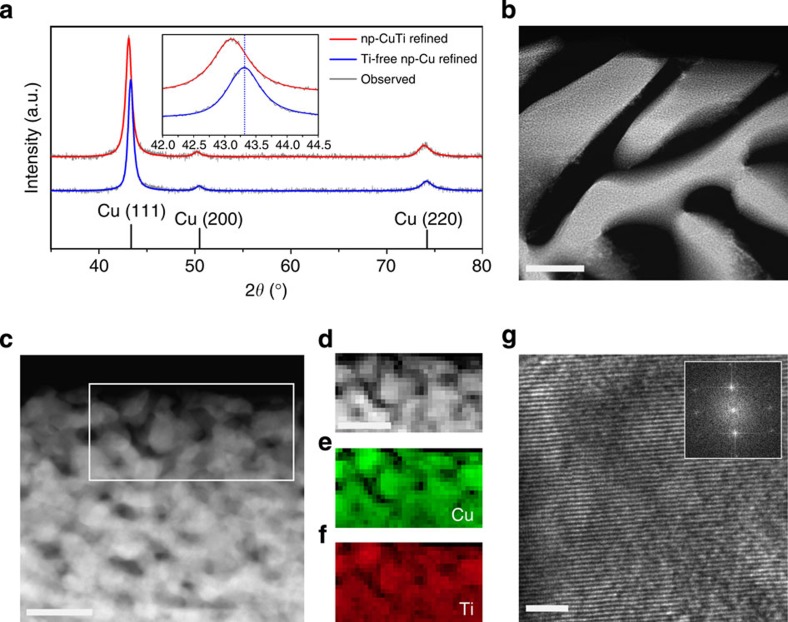
XRD and TEM characterization. (**a**) The XRD patterns of np-CuTi and Ti-free np-Cu. Inset: the enlarged region of Cu (111) diffraction peaks, with the dotted line indicating the peak position of pure Cu. (**b**) High-angle annular dark-field (HAADF) scanning (S)TEM image of a cross-sectioned np-CuTi sample prepared using FIB technique. Scale bar, 1 μm. (**c**), HAADF STEM image with a higher magnification. The box indicates the region selected for EELS study. Scale bar, 50 nm. (**d**–**f**) The contrast image of the selected region for EELS mapping study and its corresponding Cu (**e**) and Ti (**f**) maps. Scale bar, 50 nm. (**g**) High-resolution TEM image with visible lattice fringes. Inset: The Fourier transform confirms that np-CuTi is composed of an extended crystalline network. Scale bar, 2 nm.

The PXRD data for np-CuTi (Fig. 3a) suggested a similar crystal structure with that of the bulk Cu-Ti alloys. A small unit cell expansion (Supplementary Table 2) was also observed because ofTi substitution. The atomic ratio of Ti to (Cu+Ti) of np-CuTi was verified to be about 5 at. % by EDX analysis (Supplementary Fig. 5), mimicking the optimal composition of Cu_95_Ti_5_ from the bulk Cu-Ti alloy study. The consistency in their surface conditions was also confirmed using XPS characterizations (Supplementary Fig. 6). To further study the structure of np-CuTi, transmission electron microscopy (TEM) characterization was performed on a cross-sectioned specimen prepared using a focused ion beam (FIB) technique. High-angle annular dark-field (HAADF)-TEM image again confirmed the bimodal porous nature of np-CuTi (Fig. 3b). The high-magnification image, Fig. 3c, clearly shows that the material ligaments and nanopores are similar in size (ca 15 nm), in good agreement with the grain size estimated from PXRD data using the Scherrer’s method (Supplementary Table 2) and the pore size observed in N_2_ adsorption-desorption analysis (Supplementary Fig. 4). The np-CuTi catalyst was also examined using electron energy loss spectroscopy (EELS). Although Fig. 3d shows the contrast image of a selected region for spectroscopic evaluation, Fig. 3e,f shows the associated Cu and Ti EELS mappings using the L_2,3_ edge of Cu and Ti, respectively. It is evident that Cu and Ti atoms are homogeneously distributed along the material ligaments, consistent with the conclusion of a solid solution from the PXRD results. Moreover, near-edge fine structure analysis confirmed the metallic nature of Cu and Ti. No oxygen K-edge signal was detected in the EELS spectra. High-resolution TEM image exhibits uniform lattice fringes (Fig. 3g), further confirming the highly crystalline nature of np-CuTi.

**Figure 4:**
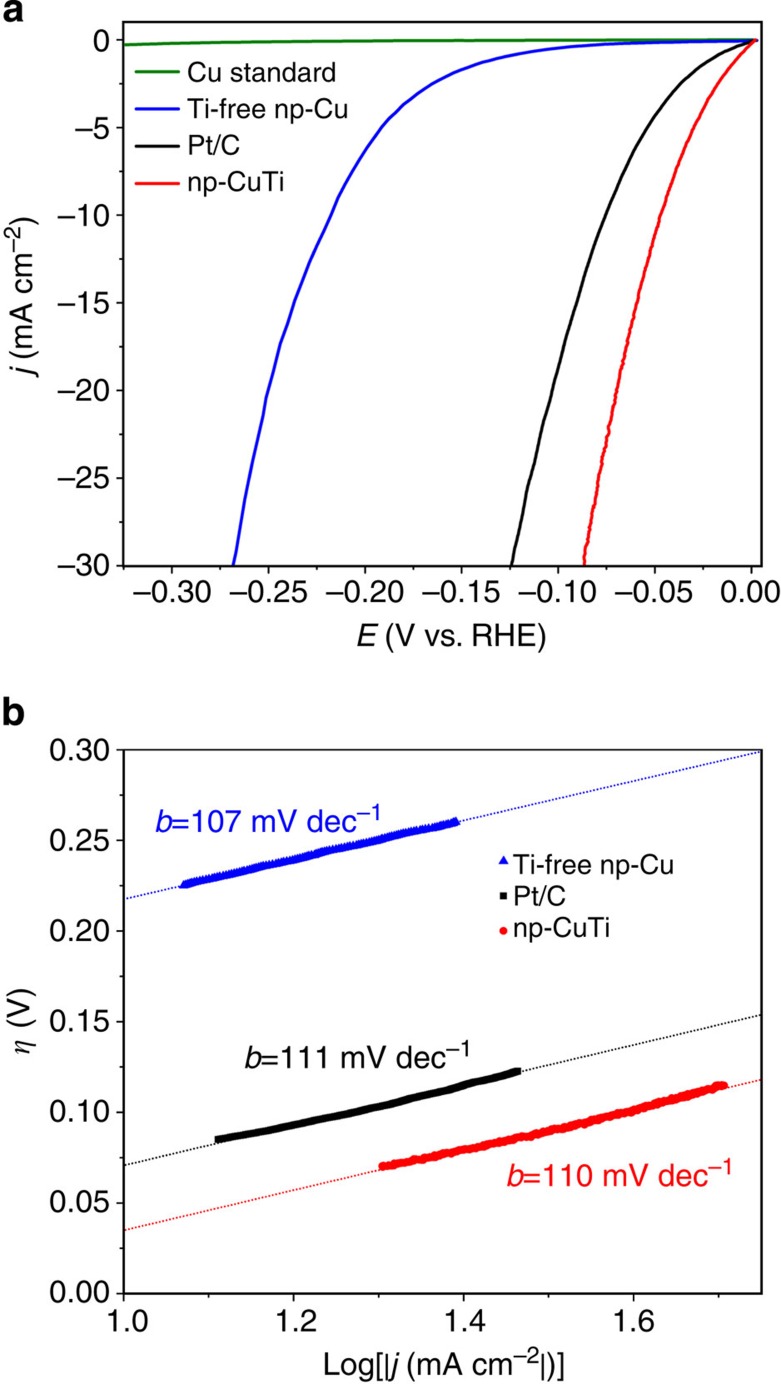
Electrochemical characterization. (**a**) HER activities for Pt/C, np-CuTi, Ti-free np-Cu control sample and polycrystalline Cu standard in 0.1 M KOH electrolyte. (**b**) The corresponding Tafel plots.

The electrocatalytic performances of np-CuTi were evaluated and compared with a commercial state-of-the-art Pt/C electrocatalyst. Figure 4a shows the HER polarization curves of normalized current densities versus applied potential (*iR* corrected). The activity of the np-CuTi catalyst exceeded Pt/C steadily with a more than twofold enhancement, most likely owing to its highly active surface, large surface area and enhanced mass transport properties. To prove the high activity of Cu-Cu-Ti sites on the internal np-CuTi surface, a Ti-free nanoporous Cu with identical hierarchical porous structure (denoted as np-Cu) was synthesized by introducing one additional dealloying process using aqueous H_2_SO_4_ solution to remove the Ti content from np-CuTi. Structural characterization results confirmed that the resulting material exhibited a near-identical structure as np-CuTi in terms of morphology (Supplementary Fig. 7), pore size distribution and specific surface area (Supplementary Fig. 1), but no Ti was detected by EDX (Supplementary Fig. 5) or XPS (Supplementary Fig. 6) analysis. More importantly, the X-ray diffraction peaks of np-Cu were shifted towards higher angles compared with those of np-CuTi and aligned precisely with the peak positions of pure Cu (Fig. 3a), indicating a successful removal of Ti atoms with a concomitant lattice contraction (Supplementary Table 2). As expected, the HER activity of the Ti-free np-Cu sample, although sharing a similar hierarchical porous structure, decreased by a factor of more than 50. It should be noted that decreasing the size of copper can lead to enhanced HER activity as can be seen that the HER activity of np-Cu is much higher than that of bulk Cu (Fig. 4a), but such an enhancement is not the dominant reason of the unique HER activity of np-CuTi. Based on the electrocatalysis results for both np-Cu and bulk polycrystalline Cu, it can be concluded that the exceptional HER activity observed in the np-CuTi sample (Fig. 4a) is due to the combination of the active Cu-Cu-Ti surface sites and the hierarchical porous structure. The long-term stability of np-CuTi catalyst was also examined with an extended reaction period of 5,000 potential cycles, in which both the electrochemical performance and material structure remained remarkably stable (Supplementary Figs 8 and 9).

## Discussion

In alkaline conditions, HER proceeds in the following sequences of either Volmer–Heyrosky or Volmer–Tafel mechanisms:


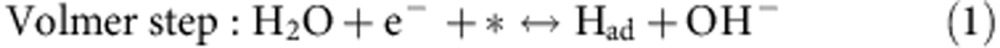



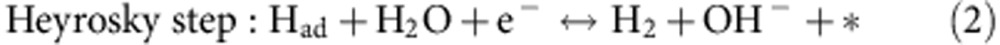



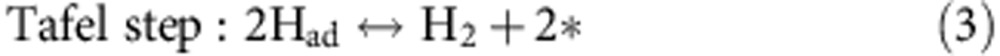


where * represents the hydrogen adsorption sites. In a recent study conducted by Durst *et al*., it is reported that the Volmer step is the rate-limiting step for HER on Pt/C in alkaline conditions, leading to a Tafel slope of about 120 mV dec^−1^ (ref. 38). Although a systematic kinetic study is beyond the scope of the current work, the Tafel analysis of np-CuTi was performed with an attempt to gain insights into the kinetics of HER. The linear portions of the Tafel plots were fitted to the Tafel equation (*η*=*b* log|*j|*+*a*) and yielded the Tafel slope (*b*; Fig. 4b). As shown in Fig. 4b, the np-CuTi catalyst exhibits a Tafel slope of 110 mV dec^−1^ (*η*=70–115 mV), and is very close to the value of Pt/C (111 mV dec^−1^, *η*=85–120 mV). Ideally, the Tafel slope is an inherent property of electrocatalytic materials and is a useful indicator of the rate-limiting step for reactions involving electron transfer. In reality, however, the Tafel slope can be dependent on many factors other than the kinetic exponent of the electrons, such as the presence of adsorbates and the mass transport effect in porous structure. Although the results in Fig. 4 showed nearly identical Tafel slopes for np-CuTi and Pt/C, future mechanistic studies are desired in order to determine whether the HER kinetics of np-CuTi is similar to that of Pt/C.

It is also worthnoting that partial surface oxidation of both Cu and Ti were observed from np-CuTi (Supplementary Fig. 6). Although the HER is known to provide a highly reductive environment, the exact surface chemical nature of np-CuTi under reaction conditions was not clear because of the lack of *in situ* methods capable of resolving the valence state of surface atoms in the potential region of HER^11^ and the limitation of *ex situ* measurement (that is, extensive exposure of samples to air). It is likely that the electrode surface is not completely metallic and the Ti atoms may also be in an oxidized state of Ti^3+^ or Ti^4+^ irrespective of contact of oxygen. It is reported recently that oxophilic groups, such as non-precious metal hydroxides, are able to aid metallic catalysts during their HER processes in alkaline because of their ability to break the O–H bond^11^,^13^,^52^,^53^. Therefore, there is a plausible scenario that un-reduced surface oxides (if there is any) of np-CuTi would facilitate the HER in a similar manner, and therefore could explain the enhanced HER activity in addition to the optimal HBE suggested by the DFT results.

In summary, a new non-precious bimetallic material, Cu-Ti alloy, was identified to be highly active for hydrogen evolution reaction under basic conditions through DFT predictions and experimental verification. A hierarchical porous Cu_95_Ti_5_ catalyst with a bimodal pore size distribution was designed and synthesized for the first time, which exhibited an HER activity more than twice that of the state-of-the-art Pt/C electrocatalyst. The achieved high activity, high stability and cost-effectiveness make np-CuTi a most promising electrocatalyst with overall HER performance. An important area of future studies is to determine the surface valence state using *in situ*/*operando* surface-sensitive techniques. Also, a scale-up test of np-CuTi using practical electrolysers, such as hydroxide exchange membrane-based electrolysers, is highly desired for the implementation to commercial processes.

## Methods

### DFT calculations

The DFT calculation of HBE was performed with the Vienna *Ab initio* Simulation Package. The PW-91 function was used in the generalized gradient approximation calculation, and a kinetic cutoff energy of 396 eV was used for the plane wave truncation. A periodic 3 × 3 unit cell with a 3 × 3 × 1 Monkhorst-Pack k-point grid was used for all calculations. All surfaces were modelled by adding six equivalent layers of vacuum onto four layers of metal atoms corresponding to the most close-packed configurations. The two bottom layers of the slab were fixed at a distance of 2.59 Å, whereas the top two layers were allowed to relax to reach the lowest energy configuration. Spin-polarization was included for both surfaces. The binding energy was calculated using the equation:


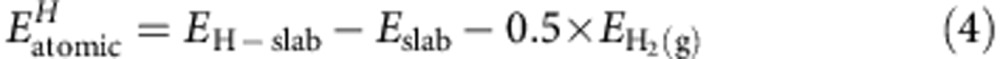


Where 
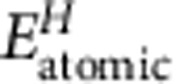
 is the binding energy of atomic hydrogen on the given slab, *E*_*H–slab*_ is the energy of the surface with 1/9 ML hydrogen adsorbed, *E*_slab_ is the energy of the slab in a vacuum and 
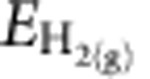
 is the energy of hydrogen in the gas phase. Different adsorption sites, such as atop, bridge, *fcc* and *hcp*, were calculated. The hcp sites always yield the lowest adsorption energy, indicating they are the most stable binding site and their values are included in Fig. 1b. The inclusion of a Ti atom in the sublayer of the model only slightly affect the calculated HBE value and does not influence the HBE trend (Supplementary Table 3).

### Materials

The Cu_100−*x*_Ti_*x*_ (*x*=1, 3, 5, 7 and 9) alloys with nominal compositions were prepared by arc melting pure Cu (Alfa Aesar, 99.999%) and Ti (Alfa Aesar, 99.99%) under an argon atmosphere. In a subsequent step, a melt spinning technique was introduced to re-melt the alloy ingot and rapidly quench on the surface of a spinning metal roller (50 m s^−1^) to achieve a homogeneous Cu(Ti) solution phase (Supplementary Fig. 1). The resulting alloy materials were in a ribbon form with a dimension about 3 mm wide and 0.5 m long. After their compositions were verified using energy dispersive X-ray spectroscopy, the alloy ribbon surfaces were polished using 0.1, 0.05 and 0.01 μm size alumina particles (Buehler) in sequence. Electrodes for electrochemical testing were fabricated by attaching one end of those alloy ribbons with copper wire (Alfa Aesar, 99.999%) as the current collector using silver paint (SPI Supplies). The apparent electrode size used for hydrogen evolution test is about 0.30 cm^2^. For the preparation of Pt/C catalyst electrode, 10 mg commercial Pt/C catalyst (TKK, 48.8 wt%) was dispensed in 20 ml deionized water. After a rigorous sonication of 30 min in a water/ice mixture bath, 15 μl of the suspension was deposited onto a glassy carbon electrode (0.20 cm^2^) and was dried in air to form a uniform thin film for electrochemical characterizations.

To synthesize hierarchical porous Cu-Ti catalyst, an Al-Cu-Ti precursor was first prepared by arc melting pure Al (Alfa Aesar, 99.99%), Cu (Alfa Aesar, 99.999%) and Ti (Alfa Aesar, 99.99%) with desired atomic ratio (80:19:1) under an argon atmosphere. After the verification of composition by energy dispersive X-ray spectroscopy, the resulting alloy ingot was cut into thin plates with dimensions of 10 × 5 × 0.20 mm^3^ using a precision wafering machine. The surface rust was removed using 240 Grit sandpaper, and the surface was further polished using finer grade sandpapers (600 Grit and 1200 Grit). A copper wire, which served as the current collector, was connected to the one end of the alloy plate using spot welding. In a following step, the pristine electrodes were immersed into a 6 M KOH solution to remove Al with a free corrosion-forming hierarchical porous Cu-Ti. For further removing Ti, the Cu-Ti catalyst was dealloyed for a second time in a 0.05 M H_2_SO_4_ solution for about 10 min until no gas bubbles were produced from the materials. Those resulting catalysts were rinsed in DI water for multiple times and applied to the electrochemical evaluations directly without drying. The apparent electrode size of hierarchical porous catalysts used for hydrogen evolution test is about 0.50 cm^2^.

### Structural characterization

PXRD patterns were collected using a Rigaku Ultima IV X-ray diffractometer with Cu Kα radiation. The porous material samples were assembled in an Ar-filled glove box with a Mylar film (Chemplex, 2.5 μm thick) mounted on the surface for preventing severe oxidation. Refinement of the PXRD patterns was conducted using the Rietveld approach implemented in Rigaku’s software package PDXL. SEM studies were performed with a ZEISS CrossBeam Auriga 60 FIB-SEM. The high-resolution TEM (bright field) image and HAADF image were taken by 200 kV FEI F20 UT Tecnai with spatial resolution of 0.14 nm and an energy resolution of EELS of 0.6 eV without a monochromator. The energy dispersion for EELS mapping was set to 0.3 eV per channel and the acquisition time for each spectrum was set at 1.2 s to achieve decent signal for Ti, Cu and O. To obtain high-spatial resolution for EELS, the total acquisition time for each map was set to be at least 40 min, which corresponds to around 1,200 pixels with drift correction. The EELS mappings are extracted from target element’s peak independently and do not reflect the relative proportion between Cu and Ti. The cross-sectioned TEM sample was prepared with the ZEISS CrossBeam Auriga 60 FIB-SEM. The porous material was embedded in M-Bond 610 Adhesive System (SPI Supplies) for improving mechanical properties before the FIB preparation. The surface roughness factors of bulk Cu-Ti alloys were characterized using an atomic force microscopy (Dimension 3100, Veeco instruments Inc.) in tapping mode (Supplementary Fig. 10). Six different areas (25 × 25 μm^2^) were randomly selected for each samples and were analysed with same scan parameters and same scan rate of 1 Hz. The roughness factors listed in Supplementary Table 1 were the average value from six different measurements. An XPS system (Physical Electronics VersaProbe 5000) was used to analyse the surface property. The system is equipped with a 16-channel hemispherical analyser and Al anode monochromatic X-ray source. The binding energy scale was calibrated by comparing the position of the primary photoelectron peaks in Cu, Au and Ag reference foils to values in literature. Data were analysed using CasaXPS software, and peaks were fit using a Gaussian/Lorentzian product line shape and Shirley background. N_2_ adsorption/desorption isotherms were collected at 77 K by using a Micromeritics ASAP 2020.

### Electrochemical evaluation

A typical three-electrode cell equipped with an Ag/AgCl reference electrode (3.0 M KCl, BASi) was used for hydrogen evolution reaction studies. A graphite rod (Sigma-Aldrich, 99.999%) was used as counter electrode for testing Cu-Ti samples. A piece of Pt wire was used as counter electrode for testing Pt/C samples. The electrolyte was 0.1 M KOH (Sigma-Aldrich, 99.99%) made with MilliQ water (18.2 MΩ) and was continuously purged with N_2_ (Keen, 99.999%). The reference electrode was calibrated to the reversible hydrogen potential using platinum wires for both working and counter electrodes in the same electrolyte purged with H_2_ (Keen, 99.999%). The calibration resulted in a shift of −0.974 V versus the reversible hydrogen electrode (RHE). The sweep rates used in the cyclic voltammetry studies were 5 mV s^−1^ for bulk materials and Pt/C; 0.5 mV s^−1^ for porous materials in order to suppress the capacitive current due to their high surface area. All experiments were conducted using a Princeton Applied Research VersaSTAT 3 potentiostat and were performed at room temperature.

## Author contributions

Q.L. and Y.Z. fabricated the material and performed the structural characterization; Q.L. and J.R. performed the catalytic investigations; G.S.H., R.T. and Q.L. performed electron microscopy analysis; R.V.F. and Q.L. did the XPS measurement; B.T.Y. did the N_2_ adsorption/desorption measurement; Z.C. did the AFM measurement; W.Y. did the DFT calculation; Q.L., F.J. and J.G.C. designed the project; Q.L., J.Q.X., H.Z., F.J. and J.G.C. analysed and interpreted the data; Q.L., F.J. and J.G.C. wrote the manuscript; F.J. and J.G.C. supervised the whole project.

## Additional information

**How to cite this article:** Lu, Q. *et al*. Highly porous non-precious bimetallic electrocatalysts for efficient hydrogen evolution. *Nat. Commun.* 6:6567 doi: 10.1038/ncomms7567 (2015).

## Supplementary Material

Supplementary Information Supplementary Figures 1-10, Supplementary Tables 1-3 and Supplementary References

## References

[b1] Bar-Even A., Noor E., Lewis N. E. & Milo R. Design and analysis of synthetic carbon fixation pathways. Proc. Natl Acad. Sci. USA 107, 8889–8894 (2010) . 20410460 10.1073/pnas.0907176107 PMC2889323

[b2] Poizot P. & Dolhem F. Clean energy new deal for a sustainable world: from non-CO_2_ generating energy sources to greener electrochemical storage devices. Energy Environ. Sci. 4, 2003–2019 (2011) .

[b3] Quadrelli E. A., Centi G., Duplan J. L. & Perathoner S. Carbon dioxide recycling: emerging large-scale technologies with industrial potential. Chemsuschem 4, 1194–1215 (2011) . 21922677 10.1002/cssc.201100473

[b4] Hoffert M. I. . Advanced technology paths to global climate stability: Energy for a greenhouse planet. Science 298, 981–987 (2002) . 12411695 10.1126/science.1072357

[b5] Turner J. A. Sustainable hydrogen production. Science 305, 972–974 (2004) . 15310892 10.1126/science.1103197

[b6] Bhavsar S., Najera M., Solunke R. & Veser G. Chemical looping: To combustion and beyond. Catal. Today 228, 96–105 (2014) .

[b7] Yan W. & Hoekman S. K. Production of CO_2_-Free Hydrogen From Methane Dissociation: A Review. Environ. Progress Sustainable Energy 33, 213–219 (2014) .

[b8] Lewis N. S. & Nocera D. G. Powering the planet: chemical challenges in solar energy utilization. Proc. Natl Acad. Sci. USA 103, 15729–15735 (2006) . 17043226 10.1073/pnas.0603395103 PMC1635072

[b9] Sivula K., Le Formal F. & Gratzel M. Solar water splitting: progress using hematite (alpha-Fe_2_O_3_) photoelectrodes. Chemsuschem 4, 432–449 (2011) . 21416621 10.1002/cssc.201000416

[b10] Porosoff M. D., Yang X. F., Boscoboinik J. A. & Chen J. G. Molybdenum carbide as alternative catalysts to precious metals for highly selective reduction of CO_2_ to CO. Angew. Chem. Int. Ed. 53, 6705–6709 (2014) . 10.1002/anie.201404109 24839958

[b11] Danilovic N. . Enhancing the alkaline hydrogen evolution reaction activity through the bifunctionality of Ni(OH)_2_/metal catalysts. Angew. Chem. Int. Ed. 51, 12495–12498 (2012) . 10.1002/anie.201204842 23129151

[b12] Sheng W. C., Gasteiger H. A. & Shao-Horn Y. Hydrogen oxidation and evolution reaction kinetics on platinum: acid vs alkaline electrolytes. J. Electrochem. Soc. 157, B1529–B1536 (2010) .

[b13] Subbaraman R. . Enhancing hydrogen evolution activity in water splitting by tailoring Li^+^-Ni(OH)_2_-Pt interfaces. Science 334, 1256–1260 (2011) . 22144621 10.1126/science.1211934

[b14] Suntivich J., May K. J., Gasteiger H. A., Goodenough J. B. & Shao-Horn Y. A perovskite oxide optimized for oxygen evolution catalysis from molecular orbital principles. Science 334, 1383–1385 (2011) . 22033519 10.1126/science.1212858

[b15] McCrory C. C. L., Jung S., Peters J. C. & Jaramillo T. F. Benchmarking heterogeneous electrocatalysts for the oxygen evolution reaction. J. Am. Chem. Soc. 135, 16977–16987 (2013) . 24171402 10.1021/ja407115p

[b16] Esposito D. V. & Chen J. G. G. Monolayer platinum supported on tungsten carbides as low-cost electrocatalysts: opportunities and limitations. Energy Environ. Sci. 4, 3900–3912 (2011) .

[b17] Esposito D. V., Hunt S. T., Kimmel Y. C. & Chen J. G. G. A new class of electrocatalysts for hydrogen production from water electrolysis: metal monolayers supported on low-cost transition metal carbides. J. Am. Chem. Soc. 134, 3025–3033 (2012) . 22280370 10.1021/ja208656v

[b18] Jaramillo T. F. . Identification of active edge sites for electrochemical H_2_ evolution from MoS_2_ nanocatalysts. Science 317, 100–102 (2007) . 17615351 10.1126/science.1141483

[b19] Kibsgaard J., Jaramillo T. F. & Besenbacher F. Building an appropriate active-site motif into a hydrogen-evolution catalyst with thiomolybdate [Mo_3_S_13_]^2−^ clusters. Nat. Chem. 6, 248–253 (2014) . 24557141 10.1038/nchem.1853

[b20] Wang H. . Electrochemical tuning of vertically aligned MoS_2_ nanofilms and its application in improving hydrogen evolution reaction. Proc. Natl Acad. Sci. USA 110, 19701–19706 (2013) . 24248362 10.1073/pnas.1316792110 PMC3856830

[b21] Karunadasa H. I. . A molecular MoS_2_ edge site mimic for catalytic hydrogen generation. Science 335, 698–702 (2012) . 22323816 10.1126/science.1215868

[b22] Chen W. F. . Highly active and durable nanostructured molybdenum carbide electrocatalysts for hydrogen production. Energy Environ. Sci. 6, 943–951 (2013) .

[b23] Chen W.-F. . Biomass-derived electrocatalytic composites for hydrogen evolution. Energy Environ. Sci. 6, 1818–1826 (2013) .

[b24] Liao L. . A nanoporous molybdenum carbide nanowire as an electrocatalyst for hydrogen evolution reaction. Energy Environ. Sci. 7, 387–392 (2014) .

[b25] Voiry D. . Enhanced catalytic activity in strained chemically exfoliated WS_2_ nanosheets for hydrogen evolution. Nat. Mater. 12, 850–855 (2013) . 23832127 10.1038/nmat3700

[b26] Popczun E. J. . Nanostructured nickel phosphide as an electrocatalyst for the hydrogen evolution reaction. J. Am. Chem. Soc. 135, 9267–9270 (2013) . 23763295 10.1021/ja403440e

[b27] Savadogo O. & Ndzebet E. Hydrogen evolution reaction (h.e.r.) in an acidic or basic-medium on nickel electrodeposited with PW_12_O_40_^3−^ and Cu^2+^. J. Appl. Electrochem. 23, 915–921 (1993) .

[b28] Grimaud A. . Double perovskites as a family of highly active catalysts for oxygen evolution in alkaline solution. Nat. Commun. 4, 2439 (2013) . 24042731 10.1038/ncomms3439

[b29] Zhu W. . Monodisperse Au nanoparticles for selective electrocatalytic reduction of CO_2_ to CO. J. Am. Chem. Soc. 135, 16833–16836 (2013) . 24156631 10.1021/ja409445p

[b30] Kowal A. . Ternary Pt/Rh/SnO_2_ electrocatalysts for oxidizing ethanol to CO_2_. Nat. Mater. 8, 325–330 (2009) . 19169248 10.1038/nmat2359

[b31] Zheng Y. . Hydrogen evolution by a metal-free electrocatalyst. Nat. Commun. 5, 3783 (2014) . 24769657 10.1038/ncomms4783

[b32] Sasaki K. . Highly stable Pt monolayer on PdAu nanoparticle electrocatalysts for the oxygen reduction reaction. Nat. Commun. 3, 1115 (2012) . 23047673 10.1038/ncomms2124

[b33] Greeley J., Jaramillo T. F., Bonde J., Chorkendorff I. B. & Norskov J. K. Computational high-throughput screening of electrocatalytic materials for hydrogen evolution. Nat. Mater. 5, 909–913 (2006) . 17041585 10.1038/nmat1752

[b34] Sheng W., Myint M., Chen J. G. & Yan Y. Correlating the hydrogen evolution reaction activity in alkaline electrolytes with the hydrogen binding energy on monometallic surfaces. Energy Environ. Sci. 6, 1509–1512 (2013) .

[b35] Norskov J. K. . Trends in the exchange current for hydrogen evolution. J. Electrochem. Soc. 152, J23–J26 (2005) .

[b36] Trasatti S. Work function, electronegativity, and electrochemical behavior of metals.3. Electrolytic hydrogen evolution in acid solutions. J. Electroanal. Chem. 39, 163–184 (1972) .

[b37] Strmcnik D. . Improving the hydrogen oxidation reaction rate by promotion of hydroxyl adsorption. Nat. Chem. 5, 300–306 (2013) . 23511418 10.1038/nchem.1574

[b38] Durst J. . New insights into the electrochemical hydrogen oxidation and evolution reaction mechanism. Energy Environ. Sci. 7, 2255–2260 (2014) .

[b39] Sheng W. . Correlating hydrogen oxidation/evolution reaction activity on platinum at different pH with measured hydrogen binding energy. Nat. Commun. 6, 5848 (2014) . 25569511 10.1038/ncomms6848

[b40] Williams F. L. & Nason D. Binary alloy surface compositions from bulk alloy thermodynamic data. Surf. Sci. 45, 377–408 (1974) .

[b41] Ge X. . Nanoporous metal enhanced catalytic activities of amorphous molybdenum sulfide for high-efficiency hydrogen production. Adv. Mater. 26, 3100–3104 (2014) . 24554595 10.1002/adma.201305678

[b42] Peng Z., Freunberger S. A., Chen Y. & Bruce P. G. A reversible and higher-rate Li-O_2_ battery. Science 337, 563–566 (2012) . 22821984 10.1126/science.1223985

[b43] Lu Q. . A selective and efficient electrocatalyst for carbon dioxide reduction. Nat. Commun. 5, 3242 (2014) . 24476921 10.1038/ncomms4242

[b44] Gan L., Heggen M., O’Malley R., Theobald B. & Strasser P. Understanding and controlling nanoporosity formation for improving the stability of bimetallic fuel cell catalysts. Nano Lett. 13, 1131–1138 (2013) . 23360425 10.1021/nl304488q

[b45] Oezaslan M., Hasché F. & Strasser P. PtCu_3_, PtCu and Pt_3_Cu alloy nanoparticle electrocatalysts for oxygen reduction reaction in alkaline and acidic media. J. Electrochem. Soc. 159, B444–B454 (2012) .

[b46] Kibsgaard J., Chen Z., Reinecke B. N. & Jaramillo T. F. Engineering the surface structure of MoS_2_ to preferentially expose active edge sites for electrocatalysis. Nat. Mater. 11, 963–969 (2012) . 23042413 10.1038/nmat3439

[b47] Chen Z. . Core-shell MoO_3_-MoS_2_ nanowires for hydrogen evolution: a functional design for electrocatalytic materials. Nano Lett. 11, 4168–4175 (2011) . 21894935 10.1021/nl2020476

[b48] Fujita T. . Atomic origins of the high catalytic activity of nanoporous gold. Nat. Mater. 11, 775–780 (2012) . 22886067 10.1038/nmat3391

[b49] Snyder J., Fujita T., Chen M. W. & Erlebacher J. Oxygen reduction in nanoporous metal-ionic liquid composite electrocatalysts. Nat. Mater. 9, 904–907 (2010) . 20953182 10.1038/nmat2878

[b50] Strasser P. . Lattice-strain control of the activity in dealloyed core-shell fuel cell catalysts. Nat. Chem. 2, 454–460 (2010) . 20489713 10.1038/nchem.623

[b51] Oezaslan M., Heggen M. & Strasser P. Size-dependent morphology of dealloyed bimetallic catalysts: linking the nano to the macro scale. J. Am. Chem. Soc. 134, 514–524 (2012) . 22129031 10.1021/ja2088162

[b52] Cui C. . Shape-selected bimetallic nanoparticle electrocatalysts: evolution of their atomic-scale structure, chemical composition, and electrochemical reactivity under various chemical environments. Farady Discuss. 162, 91–112 (2013) . 10.1039/c3fd20159g 24015578

[b53] Ahmadi M., Behafarid F., Cui C., Strasser P. & Cuenya B. R. Long-range segregation phenomena in shape-selected bimetallic nanoparticles: chemical state effects. ACS Nano 7, 9195–9204 (2013) . 24015721 10.1021/nn403793a

