# Supplementary material for: Highly porous non-precious bimetallic electrocatalysts for efficient hydrogen evolution
Source: Nat Commun. 2015 Mar 16;6:6567. doi: 10.1038/ncomms7567 (PMC4382682; doi:10.1038/ncomms7567)
Supplement: Supplementary Information — Supplementary Figures 1-10, Supplementary Tables 1-3 and Supplementary References [file ncomms7567-s1.pdf]

Supplementary Information:

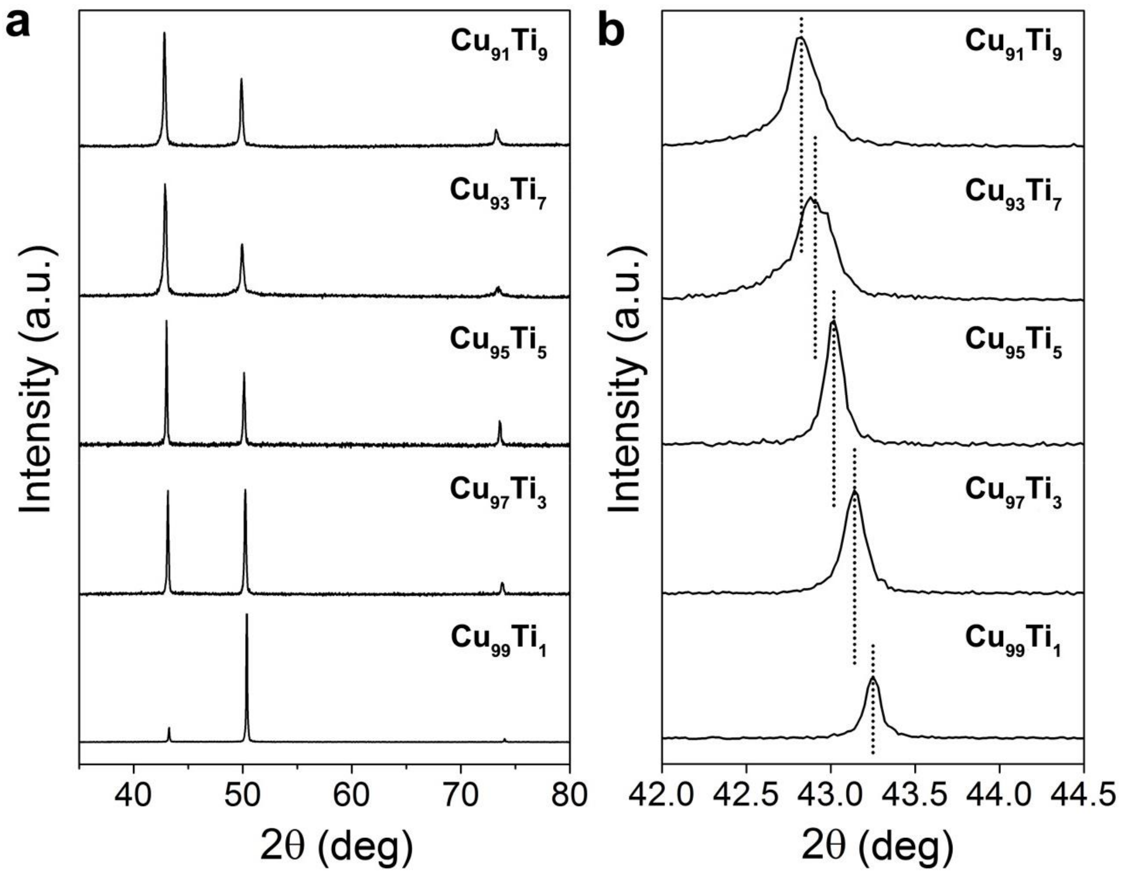

Supplementary Figure 1: PXRD characterizations for bulk Cu-Ti alloys. a, The full PXRD patterns. **b**, the enlarged Cu (111) peak region. All peaks were shifted towards to lower angular positions indicating a lattice expansion due to Ti modifications.

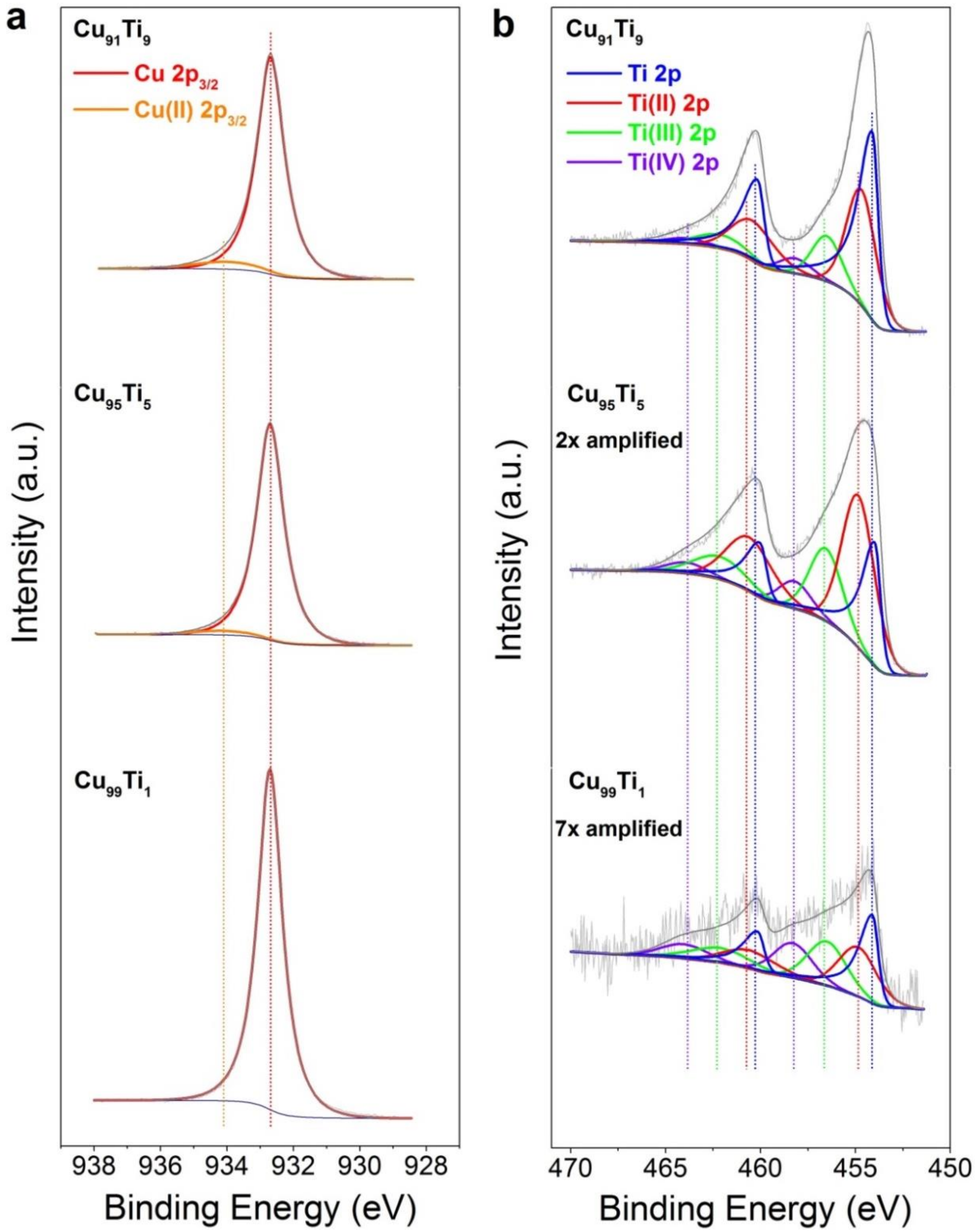

Supplementary Figure 2: XPS characterizations for bulk Cu-Ti alloys. a, Cu 2p spectra. **b**, Ti 2p spectra. The peak assignment and fitting parameters were referenced from ref (1). Partial surface oxidation was observed in both Cu and Ti due to the handling of materials in atmospheric air.

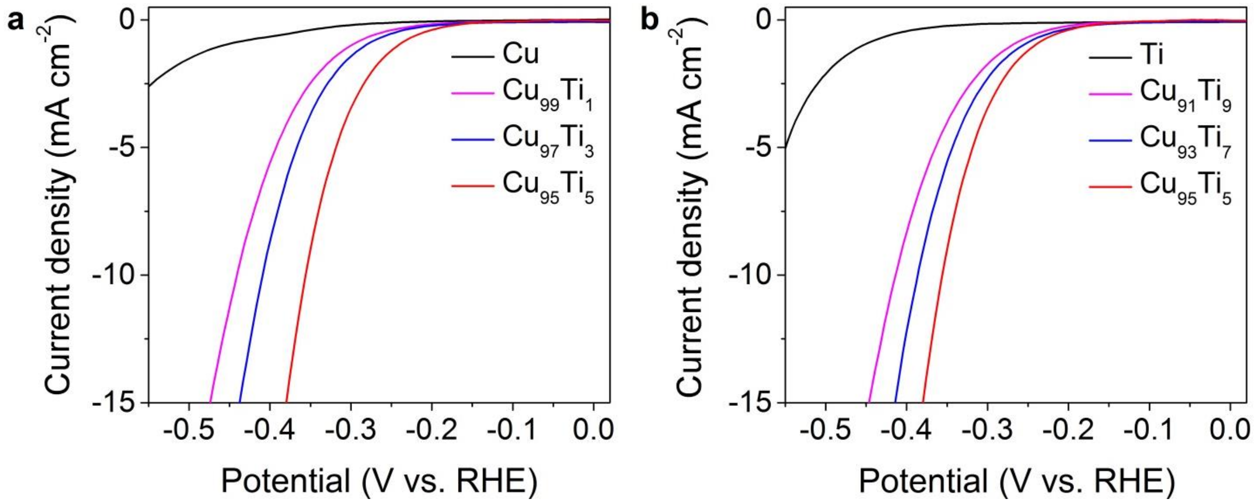

Supplementary Figure 3: HER activities of bulk Cu-Ti alloys and single metals. a, HER polarization curves for bulk Cu and Cu-Ti alloys with Ti dopings of 1, 3, and 5%. **b**, HER polarization curves for bulk Ti and Cu-Ti alloys with Ti dopings of 5, 7, and 9%. All current densities have been scaled with the surface roughness factor.

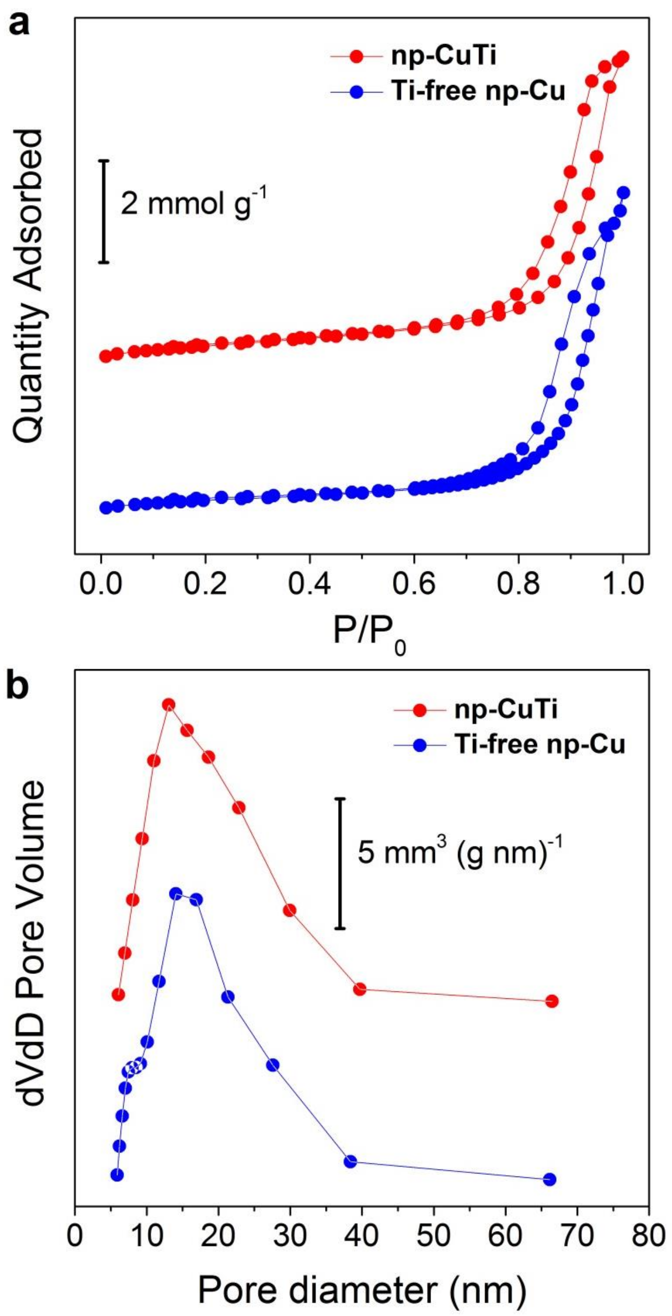

Supplementary Figure 4: N_2 adsorption/desorption characterization. **a**, The N_2 adsorption/desorption isotherms for as-prepared np-CuTi and its Ti-free np-Cu counterpart. **b**, The corresponding pore size distributions derived from the desorption isotherms using BJH method. Both materials exhibited near-identical specific surface areas (46 m^2 g^{-2} for np-CuTi; 45 m^2 g^{-2} for Ti-free np-Cu) and pore size distributions.

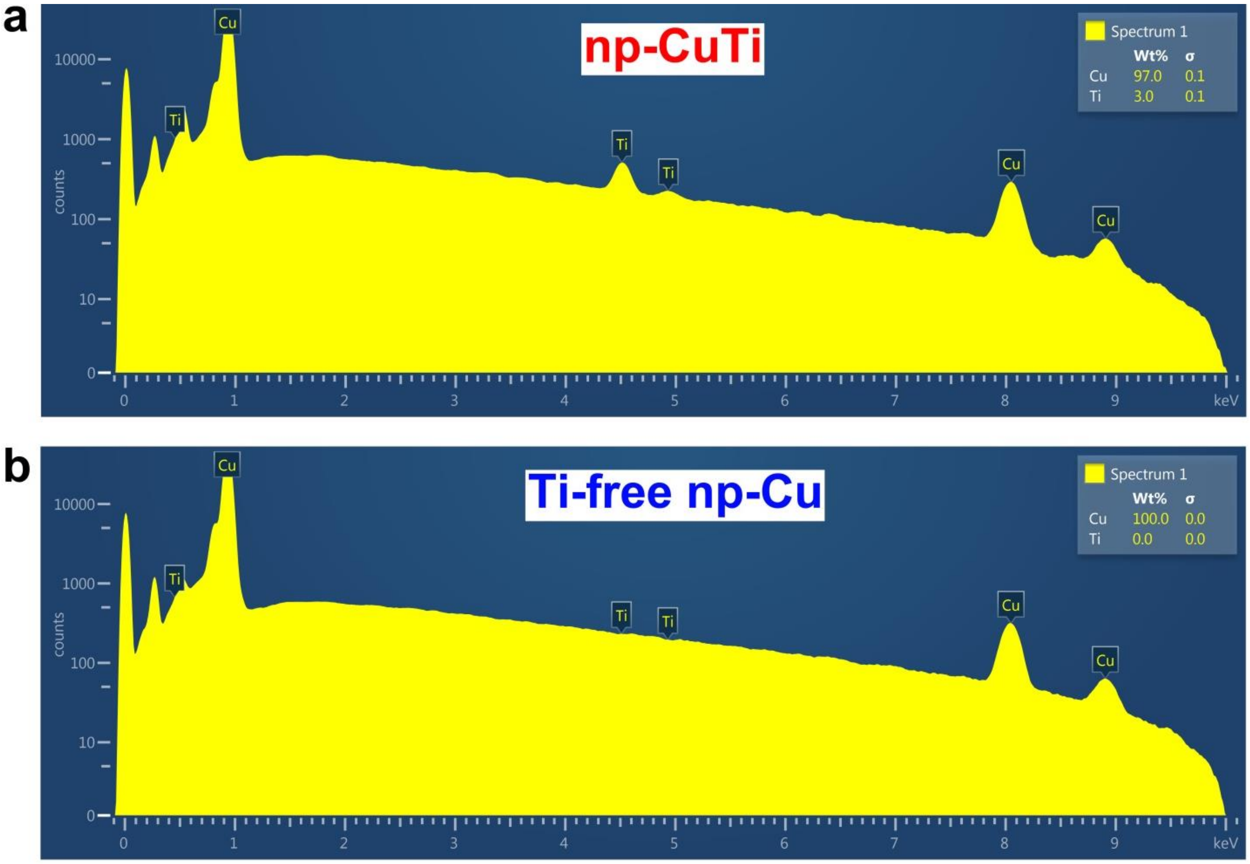

Supplementary Figure 5: EDX analysis. **a**, EDX spectrum of np-CuTi, showing a Ti concentration of about 3 wt. % (*ca.* 5 at. %). **b**, EDX spectrum of the acid-treated material showing no detectable Ti element.

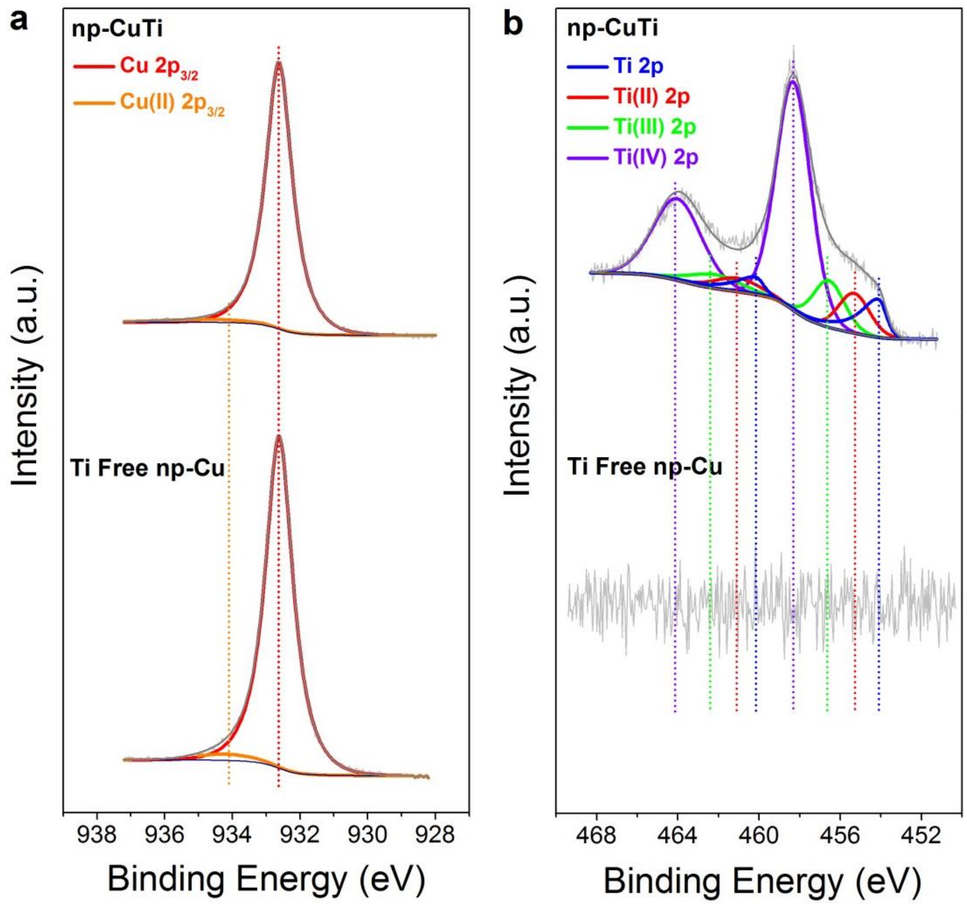

Supplementary Figure 6: XPS characterizations for np-CuTi and Ti-free np-Cu. a, Cu 2p spectra. **b**, Ti 2p spectra. The peak assignment and fitting parameters were referenced from ref (1). The surface conditions and surface Ti composition of np-CuTi is similar to that of bulk $Cu_{95}Ti_5$ (12.7% for np-CuTi; 10.9% for $Cu_{95}Ti_5$). Partial surface oxidation was observed in both Cu and Ti due to the handling of materials in atmospheric air similar as in bulk materials.

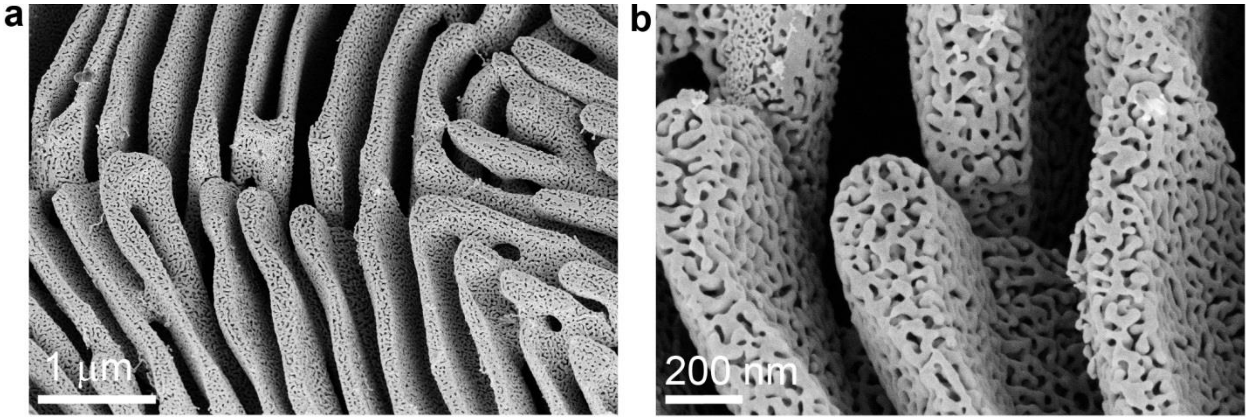

Supplementary Figure 7: SEM characterizations of Ti-free np-Cu. a, SEM image at a low magnification. b, SEM image at a higher magnification.

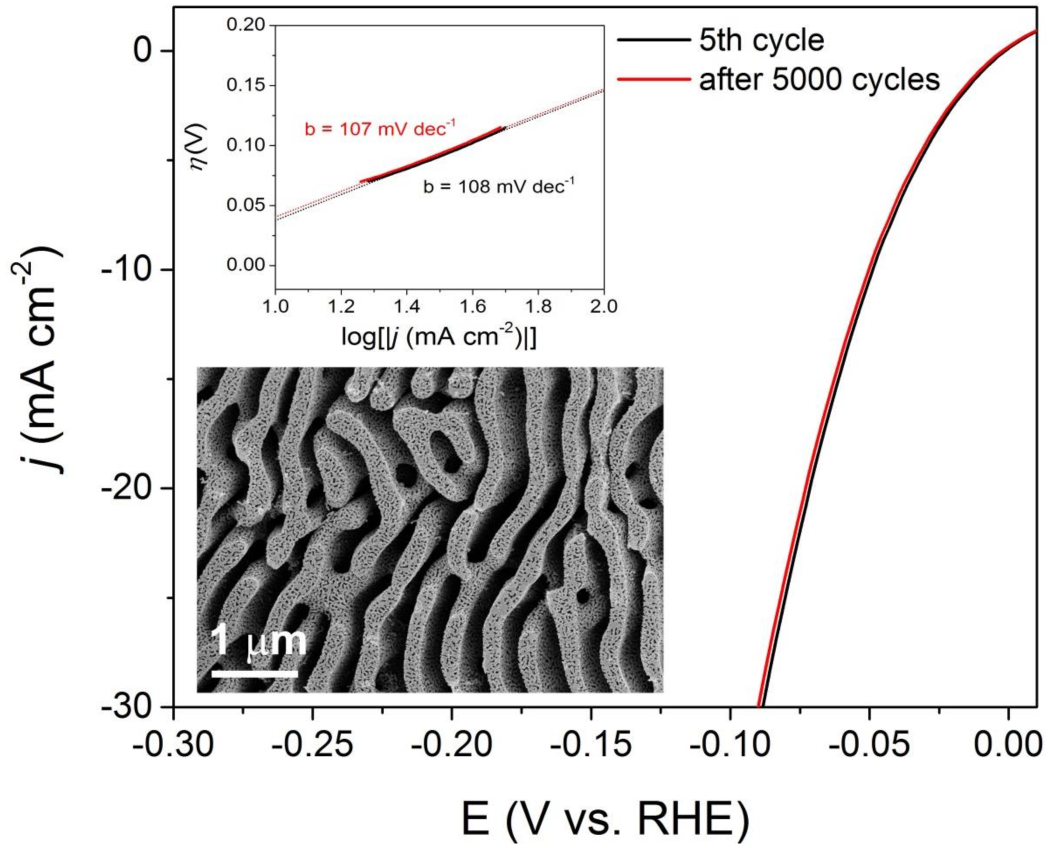

Supplementary Figure 8: Electrochemical durability of np-CuTi. HER polarization curves at the 5th cycle and after 5000 cycles with a scanning rate of 0.5 mV s^{-1}. The cycles in between were scanned at 25 mV s^{-1}. Inset: The corresponding Tafel plots (upper panel) and the morphology after 5000 cycles (lower panel).

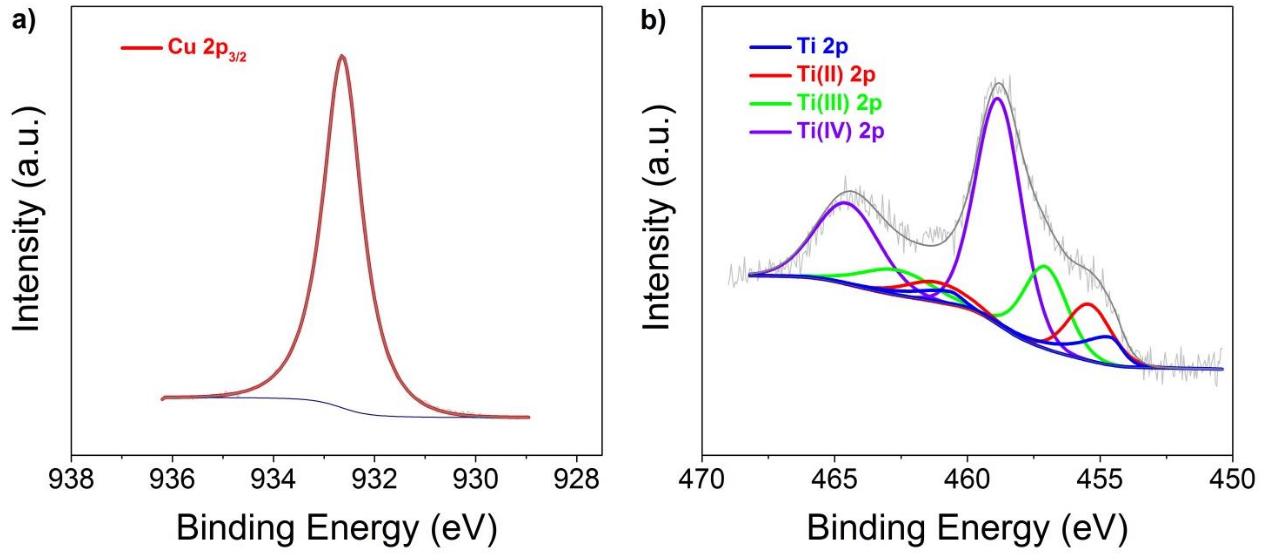

Supplementary Figure 9: XPS characterizations for post-reaction np-CuTi. XPS spectra of the post-reacted np-CuTi sample (Fig. S9) in a) Cu 2p region and b) Ti 2p region. The peak assignment and fitting parameters were referenced from ref (1). The surface condition of Ti is similar to that of an as-prepared sample. Partial surface oxidation was again observed most likely due to the handling of materials in atmospheric air similar as in bulk materials.

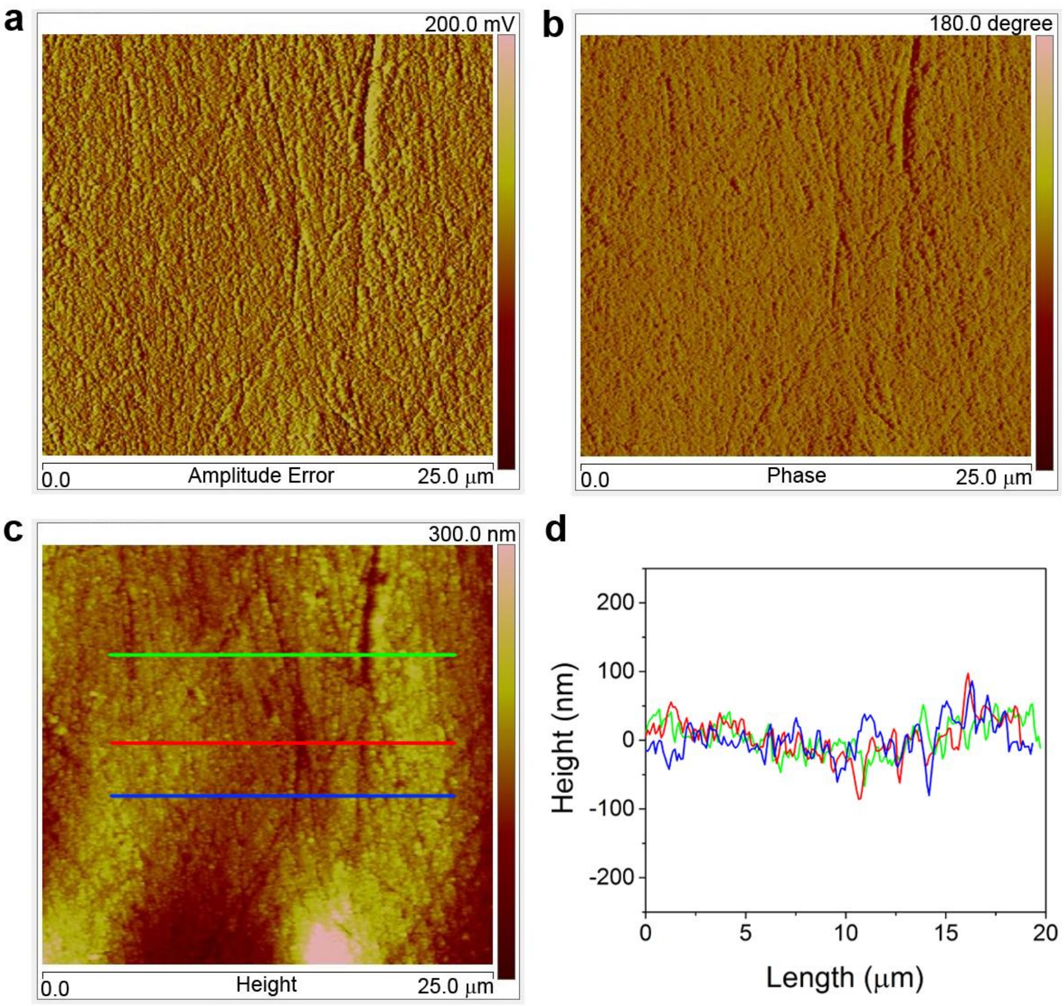

Supplementary Figure 10: Surface roughness characterization. Typical AFM images of a bulk Cu-Ti alloy surface in **a**, amplitude, **b**, phase, and **c**, height. **d**, line scans of the height fluctuations for the three lines indicated in **c**.

Supplementary Table 1: Summarized properties of bulk Cu-Ti alloys, bulk Cu and Ti standards.

Materials	j (mA cm^{-2})*	i_0 (μA cm^{-2})	Tafel Slope (mV dec^{-1})	Ti surface composition (%)	Roughness factor
Cu	0.65	0.79	139	0	1.03
$Cu_{99}Ti_1$	5.74	4.59	125	1.9	1.01
$Cu_{97}Ti_3$	8.62	8.5	123	7.3	1.01
$Cu_{95}Ti_5$	20.25	13.15	125	10.9	1.02
$Cu_{93}Ti_7$	12.26	10.44	130	15.1	1.02
$Cu_{91}Ti_9$	8.31	6.39	137	20.1	1.07
Ti	0.42	0.51	138	100	1.01

*The current densities were compared at overpotential of 0.4 V.

Supplementary Table 2: Lattice parameter and grain size estimated from PXRD pattern for np-CuTi, Ti-free np-Cu, and their comparison to theoretically constructed $Cu_{95}Ti_5$ and Cu standard.

Materials	a (Å)	b (Å)	c (Å)	V (Å^3)	Grain size (nm)
np-CuTi	3.636	3.636	3.636	48.07	13.8
Ti-free np-Cu	3.616	3.616	3.616	47.27	14.4
Constructed $Cu_{95}Ti_5$	3.634	3.634	3.634	48.00	N/A
Cu standard	3.615	3.615	3.615	47.24	N/A

Supplementary Table 3: Calculated HBE with subsurface Ti atom. A figure of the geometry at the relaxed state is provided for each case (H = white, Ti = gray, Cu = orange)

Adsorption Configuration	Hollow Site Type	Geometry	Calculated HBE (eV)
Cu-Cu-Cu	*hcp*	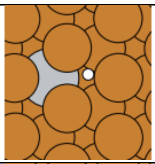	-0.09
Cu-Cu-Ti (1)	*hcp*	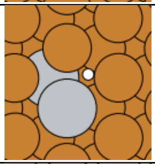	-0.42
Cu-Cu-Ti (2)	*hcp*	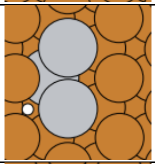	-0.40
Cu-Ti-Ti	*hcp*	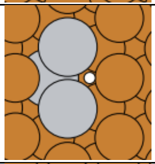	-0.81
Cu-Cu-Ti (2)	*hcp*	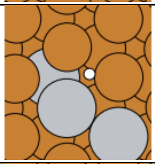	-0.34
Cu-Ti-Ti	*hcp*	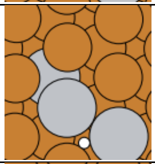	-0.74
Cu-Ti-Ti	*hcp*	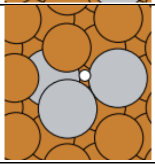	-0.80

Supplementary Reference:

1. M. C. Biesinger, L. W. M. Lau, A. R. Gerson, R. S. C. Smart, Resolving surface chemical states in XPS analysis of first row transition metals, oxides and hydroxides: Sc, Ti, V, Cu and Zn. *Applied Surface Science* **257**, 887-898 (2010).
